# Experimental constraints on light elements in the Earth’s outer core

**DOI:** 10.1038/srep22473

**Published:** 2016-03-02

**Authors:** Youjun Zhang, Toshimori Sekine, Hongliang He, Yin Yu, Fusheng Liu, Mingjian Zhang

**Affiliations:** 1 Department of Earth and Planetary Systems Science, Hiroshima University, Kagamiyama 1-3-1, Higashi-Hiroshima 739-8526, Japan; 2 National Key Laboratory of Shock Wave and Detonation Physics, Institute of Fluid Physics, China Academy of Engineering Physics, PO Box 919-111, Mianyang 621900, China; 3 College of Physical Science and Technology, Southwest Jiaotong University, Chengdu 610031, China

## Abstract

Earth’s outer core is liquid and dominantly composed of iron and nickel (~5–10 wt%). Its density, however, is ~8% lower than that of liquid iron, and requires the presence of a significant amount of light element(s). A good way to specify the light element(s) is a direct comparison of density and sound velocity measurements between seismological data and those of possible candidate compositions at the core conditions. We report the sound velocity measurements of a model core composition in the Fe-Ni-Si system at the outer core conditions by shock-wave experiments. Combining with the previous studies, we found that the best estimate for the outer core’s light elements is ~6 wt% Si, ~2 wt% S, and possible ~1–2.5 wt% O. This composition satisfies the requirements imposed by seismology, geochemistry, and some models of the early core formation. This finding may help us to further constrain the thermal structure of the Earth and the models of Earth’s core formation.

Seismological observations of the Earth’s core suggest that it occupies ~16.3 volume %, out of which 15.6% is liquid outer core and 0.7% solid inner core. Since the time F. Birch suggested that pure Fe or Fe-Ni alloy alone is too dense for the core^1^, several light elements have been proposed as candidates existing in the Earth’s core, including O, Si, S, C, and H (ref. 2). However, it has been a longstanding challenge to ascertain their varieties and concentrations because of no possibility of direct sampling and the difficulty of generating the core conditions in laboratory (extreme high pressure and high temperature). The presence of light element(s) in the core affects the Earth’s magnetic field, the dynamics of core convection, and the rate of core cooling, as well as the evolution of core segregation during the Earth formation and subsequent recrystallization of the inner core^3^,^4^.

Due to high volatilities of C and H, they are unlikely to massively incorporate into Fe at the early differentiation of the core formation^5^. So Si, S, and O are traditionally regarded as the most likely candidates that attract increasing attentions and arguments over the last two decades^6^. S is a strongly siderophile, but moderately volatile element. Geo- and cosmochemical constraints give an upper limit of ~2 wt% S in the core through mass balance calculations of the bulk Earth compositions^5^,^7^. For oxygen and silicon, besides geo- and cosmochemical arguments^5^,^8^, their presence is supported by possible chemical interactions between liquid iron and silicates at the core-mantle boundary during accretion and present^9^,^10^,^11^. Additionally, O may be the likely element that would be expelled from the recrystallized inner core and accumulated in the outer core due to its high incompatibility with solid iron inferred by *ab initio* calculations^12^. This explanation is consistent with the observed density jump across the inner-core boundary.

Si in the core has been strongly supported by the observed isotope differences between terrestrial samples and meteorites, that suggest Si was dissolved into iron during the core formation^13^,^14^. Liquid Fe-silicate equilibrium experiments also suggest that simultaneous solubilities of Si and O in liquid Fe increase with increasing pressures^9^, and that the presence of O in liquid Fe at high temperatures may drive more Si into the metal^15^.

In order to identify the light element(s) of the outer core more tightly, the most conventional approach is a direct comparison of density and sound velocity for possible candidates at the core conditions with seismic data such as the preliminary reference Earth model (PREM)^16^. Since it is very difficult to conduct reliable experiments on density and sound velocity for liquid (melting) samples at the core conditions, very few data are available to infer the composition of the liquid outer core. Although density measurements for liquid Fe-Ni-S and Fe-Ni-Si^17^ have been performed up to 94 GPa in laser-heated diamond anvil cells, at higher pressures, only solid samples^18^,^19^ have been investigated experimentally. Likewise compressional velocity measurements for liquid Fe alloys by static experiments are very few, only a few solid samples are measured up to ~100 GPa^20^,^21^,^22^,^23^.

Recently, density and sound velocity measurements on liquid Fe-O-S at the core conditions were reported by use of shock-wave experiments^24^, which suggested a possible O-depleted outer core so that Si or S might be required. However, the available experimental data for model systems at the core conditions are still insufficient to make direct comparisons with the observed seismological data.

Shock wave compression is a proven technique to study properties of materials throughout the entire Earth’s core conditions in the laboratory^24^. In this paper, we report a direct measurement of sound velocity at the core conditions for an Fe-Ni-Si system in both solid and liquid regimes by shock-wave experiments and the previously reported pressure-density relation^25^. Combining the present result with the previous work on liquid Fe and Fe-O-S (refs 24, 26, 27), we show the effects of Si, O, and S present in the Fe at the core conditions. The results constrain the light elements in the liquid outer core together with the current geochemical models by selecting a most probable composition.

## Experimental Results

Hypervelocity impact experiments were conducted to a pressure of ~280 GPa so as to obtain the sound velocity and melting behavior of Fe-9Ni-10Si system (81Fe9Ni10Si in weight per cent), which was proposed as a model composition of the outer core. Hugoniot equation of state was previously determined for this material having a density of 6.853 (±0.036) g/cm^3^ (ref. 25), using the impedance matching method (ref. 28). Shock velocity (*U*_*s*_) and particle velocity (*u*_*p*_) relation has been described by a linear relation: *U*_*s*_ (km/s) = 3.95 (±0.15) + 1.53 (±0.05) *u*_*p*_ (km/s) (ref. 25). In the present study, measurements were extended to sound velocities of the same Fe-9Ni-10Si material using the optical analyzer technique^24^,^27^,^29^ (see Methods). The experimental conditions and results are listed in Supplementary Table 1.

**Figure 1:**
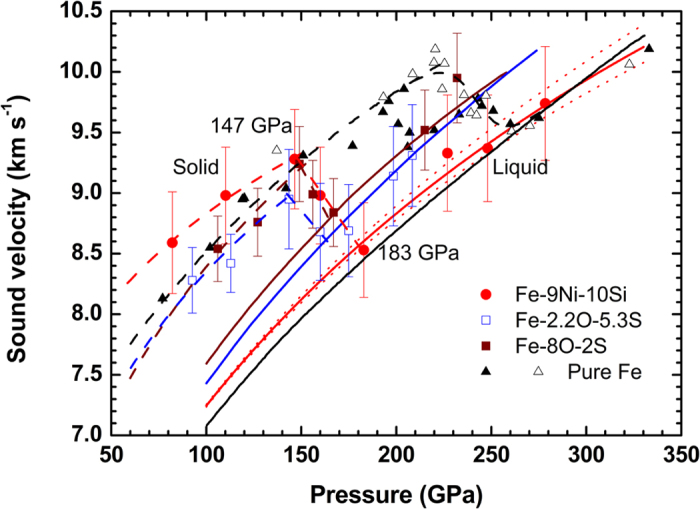
Sound velocities as a function of shock pressure for model core compositions. Solid circles for Fe-9Ni-10Si in this study; open squares for Fe-2.2O-5.3S^24^; solid squares for Fe-8O-2S^24^; solid^27^ and open^26^ triangles for pure Fe. Solid-liquid phase transition of Fe-9Ni-10Si starts at 147 ± 5 GPa (3700 ± 500 K) and completes at 183 ± 5 GPa (4150 ± 500 K). Solid red line is the bulk sound velocity of liquid Fe-9Ni-10Si calculated using a hydrodynamic and thermodynamic model^28^,^29^. Dotted red curves indicate the spread due to a 50% uncertainty in electronic contributions, which results in a ~1.6% uncertainty of sound velocity calculations at 330 GPa. Black, wine, and blue solid lines represent the calculated bulk sound velocities for three liquids: Fe, Fe-8O-2S, and Fe-2.2O-5.3S, respectively. Note that they are in good agreement with the experimental data. Dash lines are the longitudinal sound velocities at the state of solid or partial melting.

Sound velocity versus shock pressure measurements are shown in Fig. 1, and compared with previous shock data on pure Fe and other Fe-O-S systems. Abrupt changes of the sound velocity for Fe-9Ni-10Si along the Hugoniot were found at shock pressures of 147 ± 5 GPa and 183 ± 5 GPa, that were interpreted as the onset and complete melting (Fig. 1), respectively. It is noteworthy that these pressures are significantly lower than those of pure Fe (225 ± 3 GPa and 260 ± 3 GPa, respectively^26^). The sound velocity decreased by 7–8% for Fe-9Ni-10Si at the solid-melt transition. This behavior is similar to that of pure Fe (~6%)^26^. When compared to those of Fe-O-S systems^24^,^30^, the onset pressures of melting are close (144 GPa for Fe-2.2O-5.3S and 149 GPa for Fe-8O-2S, respectively), indicating that Si, O, and S have similar effects on the shock-induced melting.

**Figure 2:**
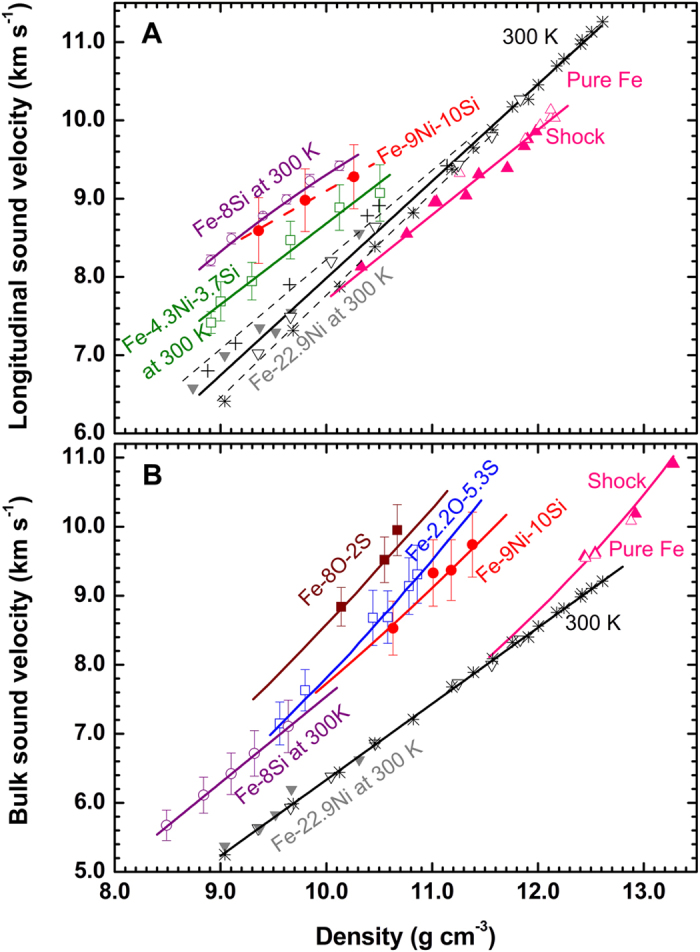
Sound velocities of pure Fe and Fe alloys. (**A**) Longitudinal sound velocities at solid states. Solid circles: Fe-9Ni-10Si from this study; open circles: Fe-8Si by static compression^23^ at 300K; open squares: Fe-4.3Ni-3.7Si by static compression^22^ at 300 K; closed inverted triangles: Fe-22.9Ni by static compression^31^ at 300 K; closed^27^ and open^26^ triangles: pure Fe by shock compression^23^, open inverted triangles^35^ and asterisks^34^: *hcp* Fe by static compression at 300 K. Fitted longitudinal sound velocities of *hcp* Fe and experimental uncertainties at 300 K are represented by black solid and dash lines, respectively. (**B**) Bulk sound velocities at liquid states and at 300 K. Closed squares: Fe-8O-2S by shock^24^; open squares: Fe-2.2O-5.3S by shock^24^; open circles: Fe-8Si by static compression^21^ at 300 K. Other symbols are same as in (**A**). Red line represents the calculated bulk sound velocity for liquid Fe-9Ni-10Si along the Hugoniot, together with those for *hcp* Fe at 300 K (black line), liquid Fe (pink line), liquid Fe-8O-2S (wine line), and liquid Fe-2.2O-5.3S (blue line).

Figure 2A,B show longitudinal sound velocity (*C*_*L*_) in solid phase and bulk sound velocity (*C*_*B*_) after melting, as a function of density (*ρ*) for various compositions. Previous studies show that the effect of Ni can be ignored on the elastic properties of Fe at the core conditions (up to ~22.9 wt%, in Fig. 2)^31^, though it can expand the stability field of body-centered cubic (*bcc*)^32^ and/or face-centered cubic (*fcc*) phases^33^ to higher pressures. As shown in Fig. 2A, Fe-9Ni-10Si has significantly faster *C*_*L*_ than both Fe^23^,^26^,^27^,^34^,^35^ in shock and static isothermal compression at 300 K and Fe-4.3Ni-3.7Si^22^ at 300 K, but slightly slower *C*_*L*_ than Fe-8Si at 300 K^23^. Temperature effect on *C*_*L*_ may be seen in the comparison of Fe data with shock^26^,^27^ and static data at 300 K^23^,^34^,^35^, indicating that *C*_*L*_ is significantly reduced at high temperatures induced in shock. Therefore, *C*_*L*_ for solid Fe-9Ni-10Si must decrease at high shock temperatures relative to the results on Fe-8Si at 300 K^23^, indicating that both the results are consistent with each other.

*C*_*B*_ of liquid Fe-9Ni-10Si along Hugoniot can be calculated as a function of pressure by a hydro- thermodynamic model^28^,^29^ (see Methods). The result reproduces well the experimental data (solid red line, Fig. 1), indicating that liquid Fe-9Ni-10Si has a slightly faster *C*_*B*_ than liquid Fe on its Hugoniot. The corresponding *C*_*B*_ *- ρ* relation for liquid Fe-9Ni-10Si is shown in Fig. 2B, and compared with the previous data for liquid Fe^26^,^27^ and Fe-O-S systems^24^ measured in shock compression. Figure 2B also shows the *C*_*B*_ of solid Fe^34^,^35^, Fe-8Si^21^ and Fe-22.9Ni^31^ in static compression at 300 K, that are derived from measured longitudinal and shear sound velocities. All the liquids of Fe-9Ni-10Si, Fe-2.2O-5.3S, and Fe-8O-2S show significantly faster *C*_*B*_ than liquid Fe as a function of density, but Fe-9Ni-10Si has a shallower slope than the Fe-O-S systems. Liquid Fe-9Ni-10Si is located between Fe-8Si at 300 K and liquid S-rich Fe-2.2O-5.3S. The differences in *C*_*B*_ and slope of *C*_*B*_/*ρ* between Fe at 300 K and liquid state of Fe in shock measurements show that *C*_*B*_ in Fe above the melting temperature increases significantly, suggesting that the *C*_*B*_ of liquid Fe-9Ni-10Si should increase in a similar fashion. Consequently, the addition of limited amounts of light elements Si, O, and/or S into liquid Fe should increase *C*_*B*_. On the other hand a comparison of *C*_*B*_ between Fe-O-S and Fe-Ni-Si liquids indicates that the presence of 8 wt% O in Fe-8O-2S increases *C*_*B*_ too much, while 10 wt% Si in Fe-9Ni-10Si and 5.3 wt% S in Fe-2.2O-5.3S may have similar effects on *C*_*B*_ at the outer core pressures.

## Discussion

In order to determine the melting curve at the core conditions based on the present shock data, we need to use the total melting condition at 183 GPa (Fig. 1). Shock temperature along the Hugoniot was not measured in our experiments but calculated by the thermodynamic relations^27^,^28^ (see Methods), which yield a temperature of 4150 ± 500 K at 183 GPa.

**Figure 3:**
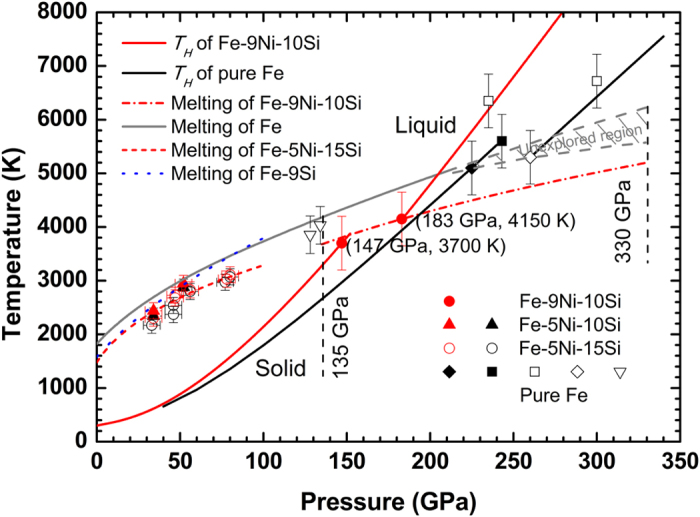
Hugoniot temperatures (*T*_*H*_) and melting curve of Fe-9Ni-10Si, compared with those of pure Fe and Fe alloys. Solid circles: melting points of Fe-9Ni-10Si from this study; solid red and black triangles: lower and upper bounds on the melting of Fe-5Ni-10Si^38^; open red and black circles: lower and upper bounds on the melting of Fe-5Ni-15Si^38^; open inverted triangles^41^, solid diamond^26^, solid^27^ and open^40^ squares: melting points of pure Fe by shock; open diamond: melting points of pure Fe^26^ by shock, taking into account the melting entropy change; solid black line: *T*_*H*_ of pure Fe^27^; solid red line: *T*_*H*_ of Fe-9Ni-10Si; dash-dotted line: melting curve of Fe-9Ni-10Si based on the Lindeman Law; solid and dash gray lines: melting curve of pure Fe^39^; dash red line: melting curve of Fe-5Ni-15Si^38^; dot blue line: melting curve of Fe-9Si^18^.

The melting curve of Fe-9Ni-10Si, *T*_*m*_(*ρ*), was modeled by the Lindeman law^36^, *d*(ln*T*_*m*_)/*d*(ln*ρ*) = 2*γ* − 2/3, using the calculated melting temperature at 183 GPa as a reference point. *γ* is the Grüneisen parameter of Fe-9Ni-10Si that includes both the lattice and electronic contributions. When extrapolated to the core-mantle boundary (CMB) and the inner core boundary (ICB), the corresponding melting temperatures are 3700 ± 500 K at 136 GPa and 5200 ± 500 K at 330 GPa, respectively. Additionally, it is known that the alloying of 5–10 wt% Ni with Fe has no significant effect on the melting temperature based on the known melting curve of Ni^37^. Thus, in combination with previously determined melting curves of Fe-9Si^18^, Fe-5Ni-10Si and Fe-5Ni-15Si^38^ (Fig. 3), the melting temperatures at pressures of CMB and ICB are estimated to be depressed by about 300–500 ± 500 K and 600–1000 ± 500 K in the presence of 10 wt% Si, respectively, compared to those of pure Fe^26^,^27^,^39^,^40^,^41^. These results are consistent with the previous study that the melting temperature of Fe is depressed by ~30 K per wt% Si at CMB and estimated by a linear interpolation^42^. The present estimation of temperature at CMB is significantly lower than those of the solidi of primitive peridotite and chondritic model mantles (above ~4150 K)^43^,^44^, but close to the recent reports on the solidi at CMB for a hydrous pyrolite (3570 ± 200 K)^45^ and a subducted basalt with volatiles (3800 ± 150 K)^46^.

Sulfur (S) has a greater effect on the depression of melting temperature of Fe than Si and O. According to the previous studies, melting temperatures for S-rich (~8–12 wt%)^38^,^47^ and O-rich (~12 wt%)^42^ core models were estimated to be ~3000 K and ~3650 K at CMB, respectively. To be consistent with the temperature at CMB, no more than 8 wt% S can exist in the outer core because such an S-rich core will make itself too cool to drive sufficient heat flow through the CMB. This interpretation supports a minor role of S and agrees with the current geochemical model (ref. 5).

**Figure 4:**
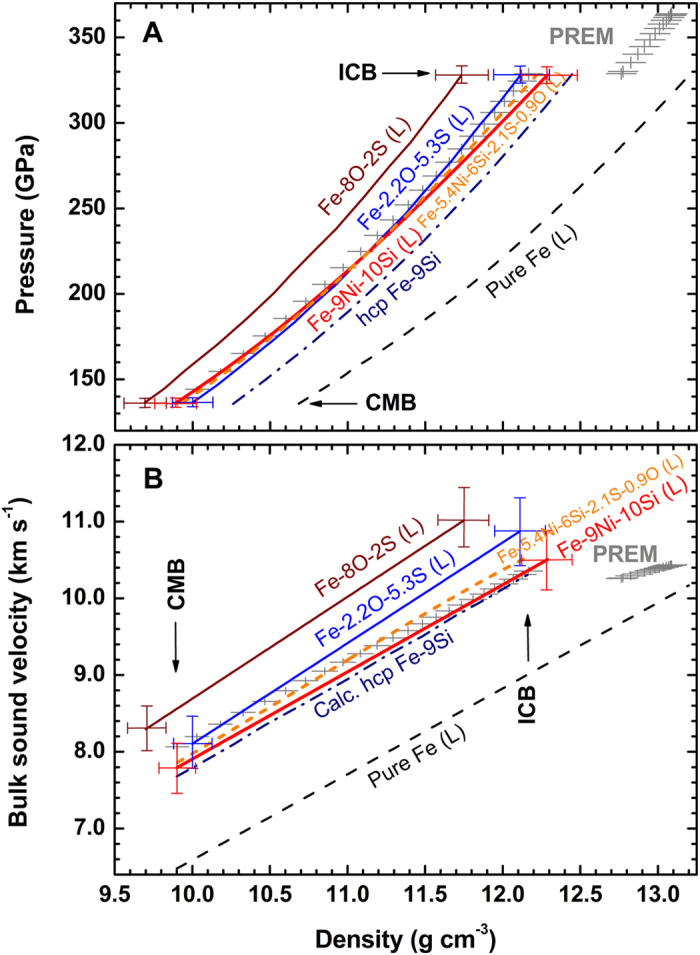
Pressure and bulk sound velocity of Fe, various Fe alloys, and PREM as a function of density at the outer core conditions. (**A**) Calculated density-pressure relations along the geotherm. (**B**) Calculated density-bulk sound velocity relations along the geotherm. Solid red line: liquid Fe-9Ni-10Si from this study; short-dash orange line: predicted liquid Fe-5.4Ni-6Si-2.1S-0.9O from this study; dash-dotted navy line: solid *hcp* Fe-9Si^18^; solid blue line: liquid Fe-2.2O-5.3S^24^; solid wine line: liquid Fe-8O-2S^24^; dash black line: liquid pure Fe^27^,^48^. PREM: preliminary reference Earth model^16^. CMB and ICB are the boundaries for the core-mantle and the outer-inner core, respectively. “L” in parentheses represents liquid state. Crosses at both ends of the lines represent the error bars of ~1–2% in density and ~4–5% error in bulk sound velocity for the Fe-Ni-Si and Fe-O-S systems.

In order to compare our results with seismological data directly, thermal corrections to the measured *C*_*B*_ were calculated using the Mie-Grüneisen theory^27^,^28^ (see Methods). Adiabatic geotherm, *T*_*g*_ = *T*_*ICB*_ (*ρ*/*ρ*_*ICB*_)^*γ*′^, is assumed for the correction where Grüneisen coefficient *γ*′ = 1.5 for the outer core^6^, density *ρ*_*ICB*_ = 12.17 g/cm^3^, and temperature *T*_*ICB*_ = 5200 K. Figure 4 shows the corrected *ρ* and *C*_*B*_ relationships for the model outer core compositions of liquid Fe^27^,^48^, liquid Fe-9Ni-10Si, liquid Fe-O-S^24^, and solid Fe-9Si^18^ in comparison with the PREM^16^ at the outer core conditions. The density profile of liquid Fe-9Ni-10Si is consistent with the PREM at the Earth’s outer core within 1% discrepancies^25^ (Fig. 4A). Its *C*_*B*_ at CMB is slower by ~3% than that of the PREM, but come closer to the PREM values at ICB (Fig. 4B). Also the *C*_*B*_ profile agrees with the corrected *C*_*B*_ of solid hexagonal closed-packed (*hcp*) Fe-9Si along the geotherm^18^ (Fig. 4B), but the densities of liquid Fe-9Ni-10Si and *hcp* Fe-9Si are different because of differences in Si content (by 1 wt%) and their phase (liquid or solid).

Regarding the densities of the model core compositions (Fig. 4A), it is clear that Fe-9Ni-10Si and Fe-2.2O-5.3S are close to the PREM at the core pressures, but Fe-8O-2S with high oxygen content is not. Also on *C*_*B*_ (Fig. 4B), PREM is found to be close to Fe-9Ni-10Si and Fe-2.2O-5.3S. Thus, it may be concluded that both a silicon-rich and a sulfur-rich core composition can match well both density and sound velocity at the outer core conditions and meet the geophysics requirements.

Additionally, if we consider the geo- and cosmochemistry^5^,^7^ constraint that S in the core is ~2 wt%, silicon will be the most dominant candidate among Si, O, and S in the outer core. Then, considering the constraints^5^,^6^,^13^ from the geochemical and Si isotope data that the core may include ~6 wt% Si and ~2 wt% S, we select 85.6Fe-5.4Ni-6Si-2.1S-0.9O (by wt%) as the most probable candidate for the outer core. This composition corresponds to a mixture of 40 wt% Fe-2.2O-5.3S^24^ and 60 wt% Fe-9Ni-10Si. As illustrated in Fig. 4, both the density and *C*_*B*_ for this composition match the seismological data over the whole range of the outer core conditions. However, the slope, d*C*_*B*_/d*ρ,* is slightly higher than the seismic observations. This may indicate a need for further research on the uncertainties in the material parameters used, possible presence of additional light elements and outer-core compositional stratification^49^.

The presence of Si in the Earth’s core suggests highly reducing conditions of the core and mantle and that Si was dissolved in liquid Fe at the time of the early core formation. However, given the current estimation of FeO content in the mantle (~8 wt%^8^,^50^), the present-day mantle is in a relatively oxidized state. Then, the reducing conditions should have gradually evolved to oxidizing conditions during the Earth’s growth. This interpretation is consistent with the proposed heterogeneous continuous accretion models that the Earth accreted initially from highly-reduced materials, but that the finally-accreted mass was more oxidized^3^,^51^,^52^. Recent metal-silicate partitioning experiments of niobium (Nb) and tantalum (Ta) also suggest a need of reducing conditions to explain the Nb/Ta ratio in the bulk silicate Earth, because lithophile elements such as Nb and Ta can become siderophile at high pressures under reducing conditions^53^. Such heterogeneous continuous accretion models predict significant amounts of Si in the core. Examples are ~6 wt% Si^51^, 4–5 wt% Si with 1.9 wt% S and possible >1 wt% O^3^, or ~8 wt% Si with ~2 wt% S and ~0.5 wt% O^52^. However, the metal-silicate partitioning data of vanadium and chromium suggest that the core could have formed under oxidizing conditions and oxygen may be an abundant light element in the core^54^. Sound velocity calculations using *ab initio* molecular dynamics^55^ suggest that oxygen (3–6 wt%) may be the dominant element to account for the density deficit in the outer core, though there is no direct experimental work to support the suggestion yet.

According to the seismological data, a marked change in density across the ICB cannot be reconciled based only on the density difference between liquid and solid. Thus, when taking into account the density jump across ICB (ref. 12) and combining the oxidizing process during the later stage of accretion (ref. 51), a proper amount of O must be considered together with Si to account for the seismic observations of the outer core. However, the previous shock wave data for liquid Fe-O-S models indicate that more than 2.5 wt% O cannot provide sufficient matches to both density and bulk sound velocity profiles of the liquid outer core (ref. 24).

In summary, we select a model for the outer core composition consisting of liquid Fe-Ni with light elements of ~6 wt% Si, ~2 wt% S, and possible ~1–2.5 wt% O. This will satisfy the requirements imposed by the density and sound velocity experiments, seismology, geochemistry, and some evolutionary models of the early core formation. Although further considerations are needed to define the chemical composition of the core, a combination of silicon and oxygen as the light elements in the outer core may be essential and plausible. In the case of a Si-rich outer core, its relatively cool geotherm at the CMB implies that water may be present at the base of lower mantle. Such a cool geotherm is in fact critical to explain the nature of the ultra-low velocity zones near the CMB, supported by recent studies^56^,^57^ on dense hydrous minerals that are stable at high pressure and high temperature (δ-phase and aluminum-rich phase D and H) and can transport water into the lowermost mantle.

## Methods

### Material

We prepared the starting material of Fe-9Ni-10Si (wt%), by sintering a mixed powder of Fe, Ni, and Si at ~1000 °C under vacuum^25^. This composition, as in the previous study^25^, was selected based on the Si-rich Fe-Ni outer core model that satisfies the geochemical and geophysical data, as well as the Si isotope data (ref. 6). Samples had a diameter of ~15.0 mm, and their average initial density is 6.853 (±0.036) g/cm^3^. Sample was analyzed using micro-X-ray diffraction techniques and electron probe microanalyzer to identify the phases and to determine their compositions, as described in the previous study^25^. Results show that the starting material contains a major phase of (Fe, Ni)_3_Si (~70 wt%), minor phases of *bcc* (Fe, Ni, Si) (~15 wt%), and *fcc* taenite (Fe, Ni) (~15 wt%). Grain sizes of these phases were less than ~200 μm, and the total oxygen was less than 0.3–0.5 wt% detected as SiO_2_. Although the starting material was heterogeneous, sample thickness (~1.0–3.5 mm) and the optical probe area (~0.6 mm in diameter) were large enough to measure averaged shock velocity and sound velocity. In addition, shock melting at high shock pressures makes the sample homogenized.

### Sound velocity measurement

The sound velocity behind the shock front for metals was obtained by an optical analyzer technique^24^,^27^,^29^. Experimental setup for sound velocity measurements of Fe-9Ni-10Si was illustrated schematically in Supplementary Fig. 1. The target had three to four steps with known thicknesses as optical analyzer positions. These optical analyzers monitor particle velocity profiles simultaneously at the analyzer points. Sample thickness at which rarefaction wave overtakes the shock wave was determined through the observed profiles. On impact, a shock wave propagates toward both backward (with speed of *U*_*f*_) and forward (with speed of *U*_*s*_) sides of the impact surface as shown in Supplementary Fig. 2. The backward-moving shock wave in the flyer is reflected as a rarefaction wave on arrival at the rear surface of the flyer and then propagates forward through the flyer (with speed of *C*_*f*_^*L*^) and to the sample (with speed of *C*_*s*_^*L*^). Because the rarefaction wave in the shocked sample moves faster than that of the forward-moving shock wave in the sample (*C*_*s*_^*L*^ > *U*_*s*_), it overtakes the shock wave in the Lagrangian coordinate system and causes a drop in the particle velocity and the shock pressure. The observed rarefaction wave velocity is either *C*_*L*_ for solid or *C*_*B*_ for liquid on Hugoniot state (ref. 29). Therefore, the decrease in sound velocity along Hugoniot indicates possible shock-induced melting, that is otherwise difficult to observe through Hugoniot measurements (*U*_*s*_-*u*_*p*_ and *P*-*ρ* relations)^29^.

Hypervelocity planar impact experiments were conducted using two-stage light-gas guns at the Institute of Fluid Physics of China Academy of Engineering Physics and Southwest Jiaotong University, China. Flyer impact velocities were measured by an electromagnetic method with an accuracy of ~0.5%. A single-crystal lithium fluoride LiF (100) coated with ~2 μm Al coating is used as the window, which is held tightly to the rear face of sample. Particle velocity histories were recorded in real time at all steps of sample-Al coating interfaces using a displacement interferometer system for any reflector simultaneously (DISAR)^58^. DISAR has a multimode fiber with a core diameter of 600 μm to transmit reflected light from a moving interface. Recorded signals with DISARs are integrated to obtain average responses from sample surfaces (600 μm in diameter). The sample diameter is large enough compared with the maximum grain size of less than 200 μm.

Four particle velocity histories were presented for shot B3 at a pressure of ~160 GPa in Supplementary Fig. 3). Supplementary Fig. 4 shows how a target thickness at which rarefaction waves overtake the shock wave was estimated by linear fitting. Time intervals for the overtake at the different steps are measured on the observed particle velocity histories.

Lagrangian sound velocities of the rarefaction wave were obtained using a velocity-distance relation,


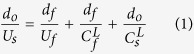


where *d*_*o*_ is overtake distance thickness; *d*_*f*_ thickness of flyer; *U*_*s*_ and *U*_*f*_ shock velocity of sample and flyer, respectively; *C*_*f*_^*L*^ and *C*_*s*_^*L*^ Lagrangian sound velocity of flyer and sample, respectively. We used pure Fe, Ta, and W flyers as described in Supplementary Table 1. When *R* = *d*_*o*_/*d*_*f*_ is defined as the ratio of overtake distance to thickness of the flyer, Lagrangian sound velocity is given by,


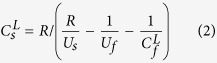


Eulerian sound velocity of sample is related to the Lagrangian velocity as follows,


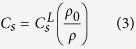


where *ρ*_*0*_ and *ρ* are initial and shocked density of sample, respectively. Uncertainty of measured sound velocities is within ~4–5%, that results from measurement and propagation errors.

There is a general relationship among the acoustic speeds: *C*_*L*_, *C*_*B*_, and transverse sound velocity (*C*_*T*_).


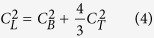


*C*_*T*_ equals to zero in liquid and gas phases, so *C*_*L*_ and *C*_*B*_ become same. A table of measured results such as impact velocity of flyer, *R*, and Eulerian sound velocity of sample along Hugoniot is given in Supplementary Table 1.

### Bulk sound velocity and shock temperature calculations

Bulk sound speed *C*_*B*_ in molten sample can be computed using the following equations^28^,^29^ along the Hugoniot pressure *P*_*H*_,


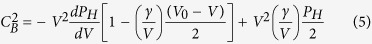



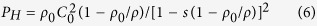


where *V*_*0*_ and *V* are volumes at the initial and shocked states, respectively. Grüneisen parameter (*γ*) in equation (5) includes lattice (*γ*_*l*_) and electronic (*γ*_*e*_)^59^ contributions,


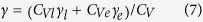



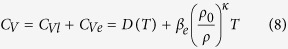


where *D*(*T*) is Debye function given by *D*(*T*) = 3*R*, *R* = the gas constant, *T* temperature, and *C*_*V*_ specific heat capacity at constant volume consisting of lattice (*C*_*Vl*_) and electronic (*C*_*Ve*_) contributions. The lattice contribution of Grüneisen parameter is described by *γ*_*l*_ = *γ*_*0*_(*ρ*_*0*_/*ρ*)^n^, where *γ*_*0*_ = 2.22 at ambient conditions and n = 1 from the measurement on Fe-9Si^18^. The electronic contribution of Grüneisen parameter is assumed same as that of *hcp* Fe^59^, i.e., *γ*_*e*_ = 2. For the electronic contribution parameter *β*_*e*_ is set to be 40 ± 20 J Mg^−1 ^K^−2^, and *κ* = 1.34, through empirical fitting to the Hugoniot data.

If samples are in thermodynamic equilibrium at shocked states, temperatures along the Hugoniot (*T*_*H*_) may be calculated from the following thermodynamic relation^27^,^28^,


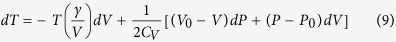


When integrated, this equation gives the temperature of 3700 ± 500 K at the measured, onset-melting pressure of 147 ± 5 GPa for Fe-9Ni-10Si. Upon completion of melting, sample *T*_*H*_ will be reduced, resulting from the latent heat of melting, by Δ*T* = *T*_*om*_Δ*S*/*C*_*V*_, where Δ*S* (melting entropy) = (0.79 ± 0.01) *R* (ref. 60), and *T*_*om*_ is the onset melting temperature at pressure of 147 ± 5 GPa.

Errors in the calculations of the bulk sound velocity and shock temperature computations come from uncertainty sources such as material parameters, especially for electronic contributions to *γ* and *C*_*V*_. Effects of the electronic contributions are shown in Supplementary Fig. 5. It shows that the calculation and the experimental results on sound velocity match. Below ~200 GPa the electronic contributions may be omitted, and the error of temperature calculations is estimated to be ~500 K based on the possible variations in Δ*S*, *γ*, *C*_*V*_, and the Hugoniot.

A non-reactive, ideal mixing model may be used to estimate the sound velocity of a composite if the sound velocity of each component was known. That is, reciprocal average sound velocity (*C*) is the sum of reciprocals of each component velocity (*C*_*i*_) weighted by volume fraction (*N*_*i*_) of each component^28^,


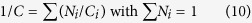


### Thermal corrections to *C*_*B*_ along the core geotherm

In order to compare the measured sound velocities with seismological data, thermal corrections to bulk sound velocities along the adiabatic core geotherm were estimated using the Mie-Grüneisen theory^27^,^28^. Thermal pressure offset (*P*_*t*_) at volume *V* is calculated by,


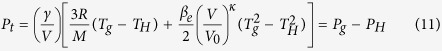


where *M* the molar mass of Fe-9Ni-10Si and adiabatic core geotherm *T*_*g*_ = *T*_*ICB*_ (*ρ*/*ρ*_*ICB*_)^*γ*^.

Then, the thermal correction to the *C*_*B*_ is calculated by,


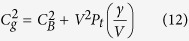


## Additional Information

**How to cite this article**: Zhang, Y. *et al.* Experimental constraints on light elements in the Earth’s outer core. *Sci. Rep.* **6**, 22473; doi: 10.1038/srep22473 (2016).

## Supplementary Material

Supplementary Information
