# Supplementary material for: Experimental constraints on light elements in the Earth’s outer core
Source: Sci Rep. 2016 Mar 2;6:22473. doi: 10.1038/srep22473 (PMC4773879; doi:10.1038/srep22473)
Supplement: Supplementary Information [file srep22473-s1.pdf]

Supplementary Material

Experimental constraints on light elements in the Earth's outer core

Youjun Zhang[1,2], Toshimori Sekine[1], Hongliang He[3], Yin Yu[3], Fusheng Liu[4], Mingjian Zhang[4]

[1]Department of Earth and Planetary Systems Science, Hiroshima University, Kagamiyama 1-3-1, Higashi-Hiroshima 739-8526, Japan.

[2]Present address: Center for High Pressure Science and Technology Advanced Research (HPSTAR), Shanghai 201900, China.

[3]National Key Laboratory of Shock Wave and Detonation Physics, Institute of Fluid Physics, China Academy of Engineering Physics, PO Box 919-111, Mianyang 621900, China.

[4]College of Physical Science and Technology, Southwest Jiaotong University, Chengdu 610031, China.

Correspondence and requests for materials should be addressed to T.S. (email: toshimori-sekine@hiroshima-u.ac.jp).

Error analysis and propagation:

We determined C_s using equations (1) – (3) in the main text:

$$C_s = R/\left(\frac{R}{U_s} - \frac{1}{U_f} - \frac{1}{C_f^L}\right)\left(\frac{\rho_0}{\rho}\right)$$

Errors in measured R and Hugoniot parameters of the sample and flyers directly affect the sound velocity measurement. Errors in C_s are estimated using the following equation:

$$\sigma_{C_s}^2 = \sigma_{\rho_0}^2\left(\frac{\partial C_s}{\partial \rho_0}\right)_{\rho_0}^2 + \sigma_{\rho}^2\left(\frac{\partial C_s}{\partial \rho}\right)_{\rho}^2 + \sigma_{R}^2\left(\frac{\partial C_s}{\partial R}\right)_{R}^2 + \sigma_{U_s}^2\left(\frac{\partial C_s}{\partial U_s}\right)_{U_s}^2 + \sigma_{U_f}^2\left(\frac{\partial C_s}{\partial U_f}\right)_{U_f}^2 + \sigma_{C_f^L}^2\left(\frac{\partial C_s}{\partial C_f^L}\right)_{C_f^L}^2$$

If we take average measuring errors to be ρ_0 (=0.5%), ρ (=1%), U_s (=2%), and R (=1.5–2%) for the current experiment, and use previously reported values of U_f (=1%) and C_f^L (=2–3%) for the standard flyer, uncertainty in C_s for our measurements is about 3.8–4.8% due to error propagation.

数式を入力します。

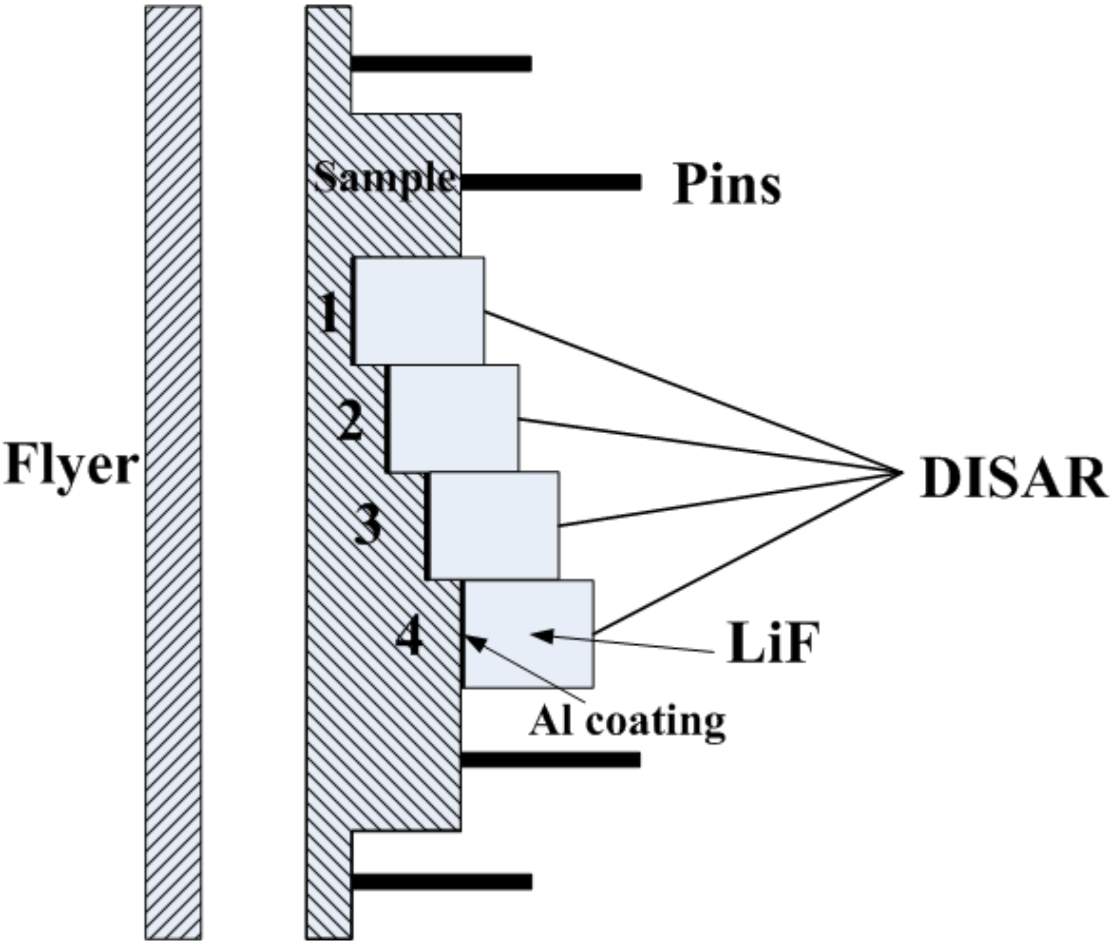

Supplementary Figure 1. Schematic diagram of experimental setup for Hugoniot and sound velocity measurements of Fe-9Ni-10Si. The target had three to four steps with a known thickness each.

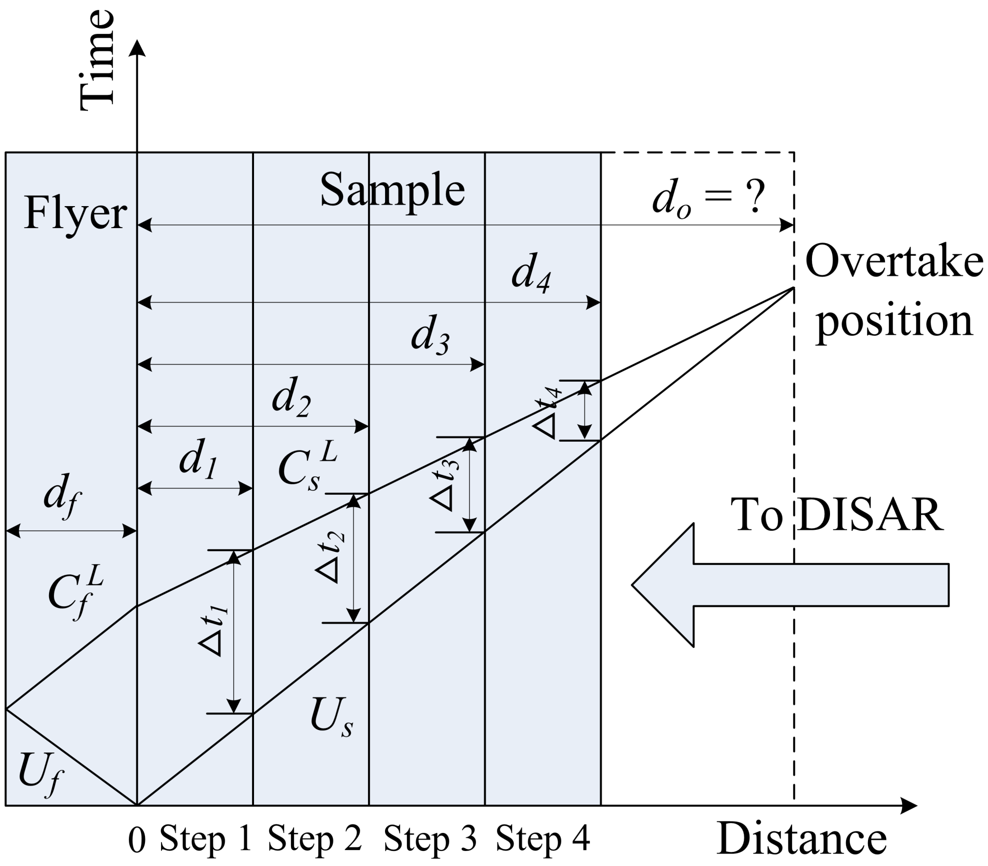

Supplementary Figure 2. Schematic Lagrangian time-distance diagram for sound velocity measurement using the optical analyzer technique by DISAR. d_1, d_2, d_3, and d_4 represent the thickness of target step 1, 2, 3, and 4, respectively; Δt_1, Δt_2, Δt_3, and Δt_4 compression durations for target step 1, 2, 3, and 4, before overtaken by the catch-up rarefaction wave, respectively; d_f thickness of the flyer; d_o overtake distance. U and C are shock and sound velocities, respectively.

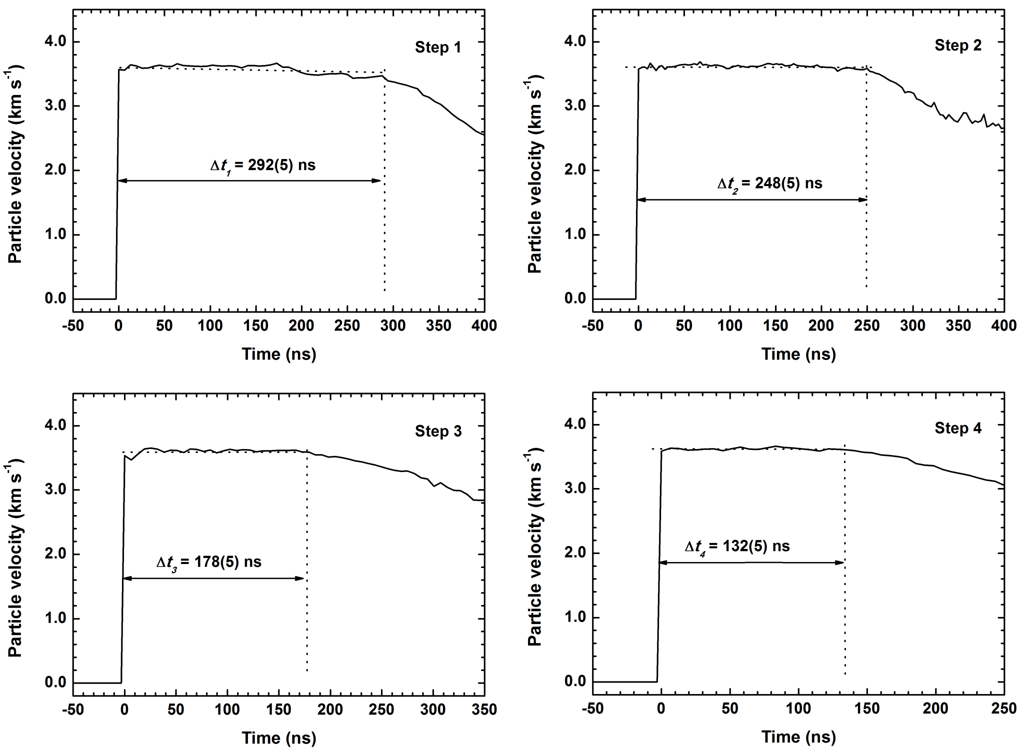

Supplementary Figure 3. Particle velocity histories for experiment shot B3 at ~160 GPa. Time intervals (292, 248, 178 and 132 ns) were determined at steps 1, 2, 3, and 4 with the thickness of 1.075, 1.812, 2.685, and 3.421 mm, respectively. Laser wavelength is 1550 nm for DISAR measurements. Particle velocities were divided by 1.267 for LiF (100) window correction (ref. 1).

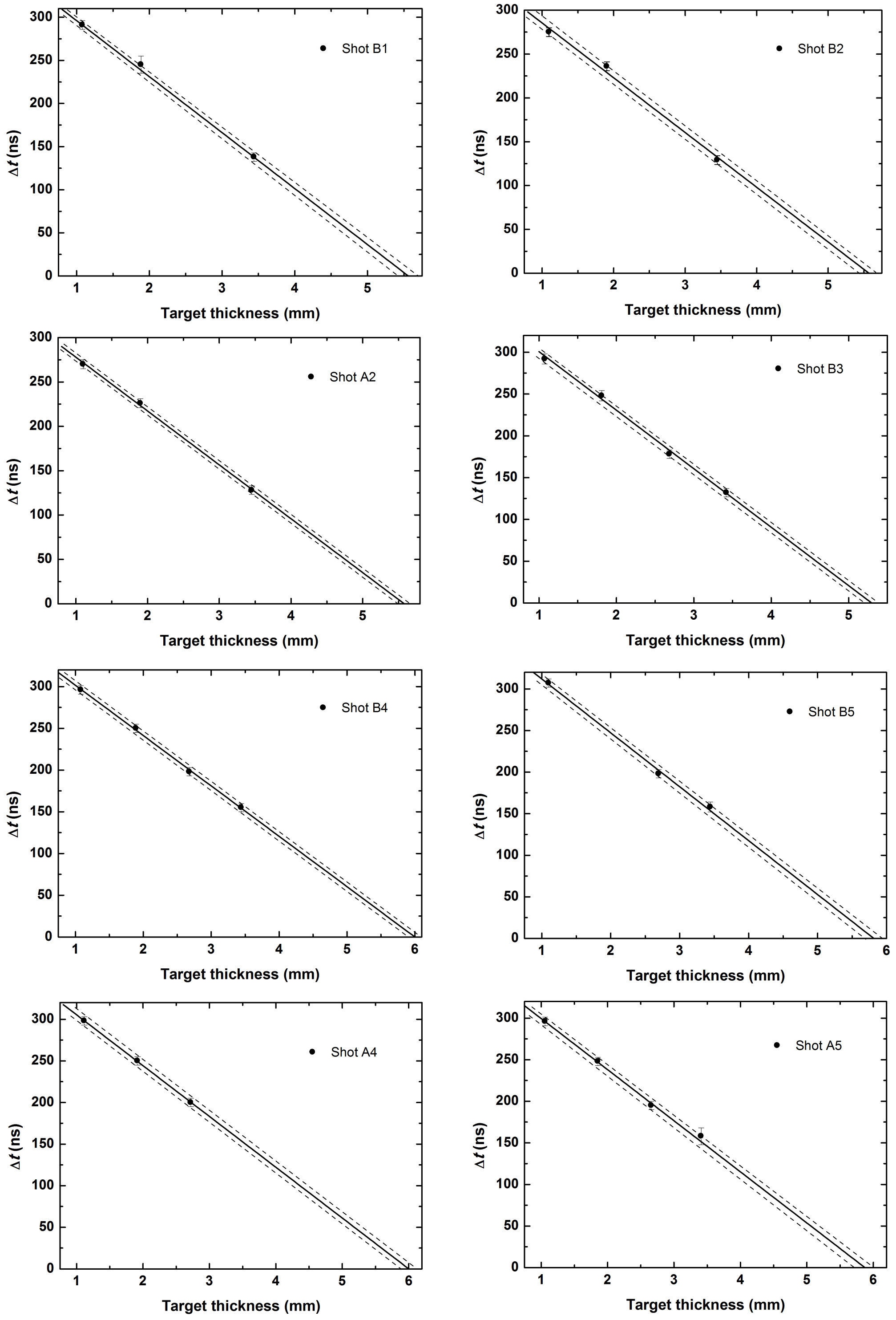

Supplementary Figure 4. Time intervals (Δt) as a function of target thickness. Intercepts of linear fits at Δt = 0 ns give overtake distance (d_o). The ratios of overtake distance to thickness of flyer (R) are listed in Supplementary Table 1.

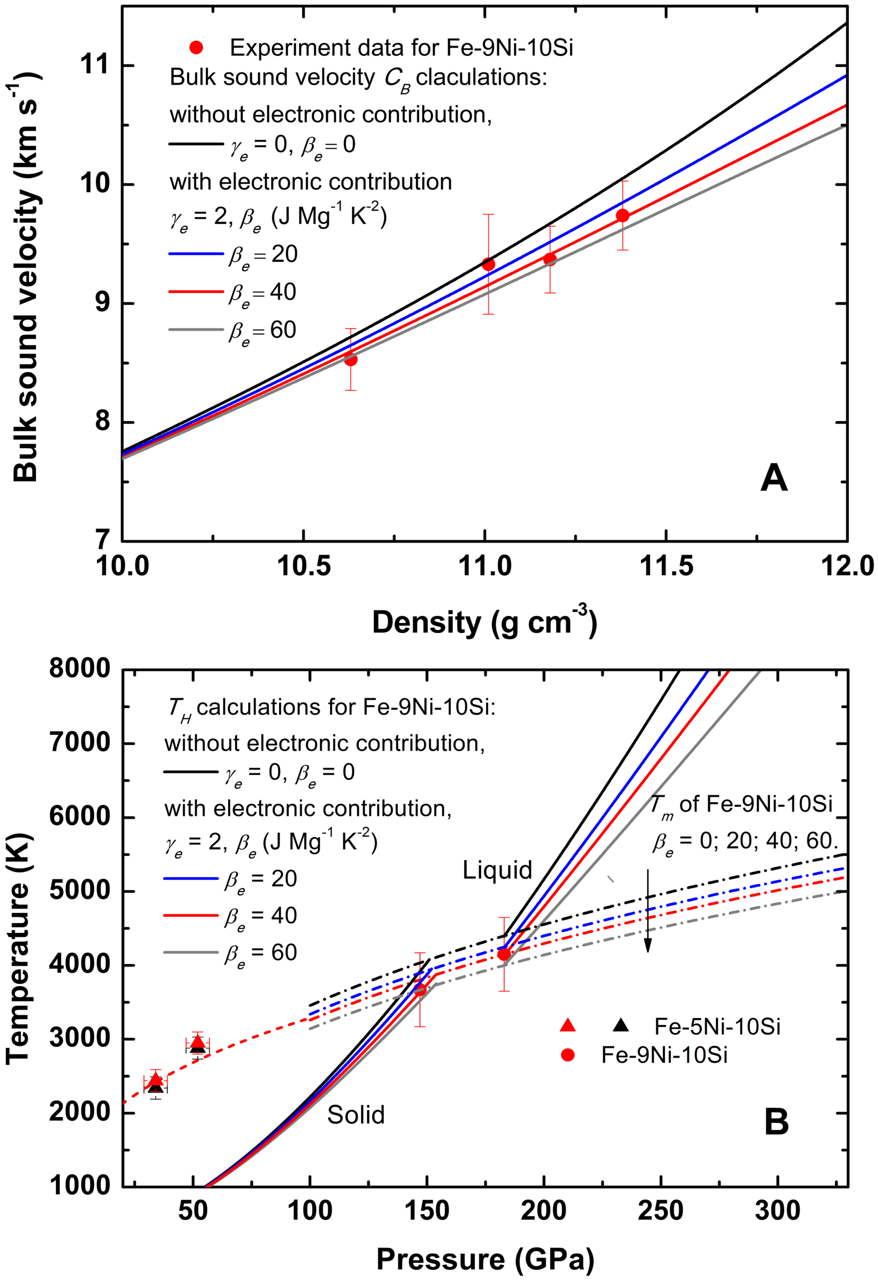

Supplementary Figure 5. Electronic contributions to the bulk sound velocity and shock temperature calculations. (A) Bulk sound velocity calculations as a function of density; **(B)** Shock temperature calculations as a function of pressure, and melting curves are deduced by the Lindeman law. Triangles represent the melting of Fe-5Ni-10Si (ref. 2). A shock-induced superheating[3] was not considered in this calculation. Below pressures of ~100 GPa, calculated bulk sound velocity differs little from that without the electronic contribution, but it differs greatly at high pressures above ~250 GPa.

Supplementary Table 1. Sound velocity measurements of Fe-9Ni-10Si*

Shot No.	Flyer (mm)	Impact velocity V_{imp} (km s^{-1})	U_s (km s^{-1})	R	Pressure (GPa)	Density (g cm^{-3})	Sound velocity (km s^{-1})
B1	1.500 Fe	3.40(0.01)	6.69(0.16)	3.70(0.06)	82.1(2.2)	9.36(0.10)	8.59(0.42)
B2	1.505 Fe	4.18(0.01)	7.31(0.17)	3.67(0.05)	110.2(2.8)	9.80(0.12)	8.98(0.40)
A2	1.503 Fe	5.07(0.01)	8.03(0.18) [7.92(0.10)]	3.72(0.06)	146.5(3.5)	10.26(0.14)	9.28(0.41)
B3	1.050 Ta	4.55(0.02)	8.27(0.18)	5.03(0.06)	159.9(4.0)	10.41(0.15)	8.98(0.40)
B4	1.050 Ta	4.98(0.02)	8.66(0.19)	5.71(0.07)	182.9(4.5)	10.63(0.16)	8.53(0.39)
B5	1.178 W	5.39(0.03)	9.36(0.20)	4.91(0.09)	226.9(5.0)	11.01(0.17)	9.33(0.48)
A4	1.190 W	5.72(0.03)	9.68(0.20) [9.73(0.16)]	5.04(0.07)	248.1(5.4)	11.18(0.17)	9.37(0.45)
A5	1.180 W	6.17(0.03)	10.10(0.21) [10.13(0.14)]	4.93(0.08)	278.4(6.0)	11.38(0.18)	9.74(0.47)

*The shock velocities, U_s, for shots B1 to B5 were calculated from the measured impact velocity of flyers, using the previously determined $U_s - u_p$ relation[4], and by the impedance matching method. The measured U_s values of shots A2, A4, and A5 from ref. 4 are listed in square brackets. Calculated values based on the same $U_s - u_p$ relation[4] are also listed and used in the present study in the same way as in shots B1 to B5. R is defined as the ratio of overtake distance in sample to flyer thickness. Errors in measurement and propagation are in parentheses. We used U_f and C_f^L along the Hugoniot of flyers for pure Fe[5,6], Ta[7,8], and W[7,9]. For Fe, initial density ρ_0 = 7.85 g/cm^3, U_s = 3.935 + 1.578 u_p (km/s); for Ta, ρ_0 = 16.68 g/cm^3, U_s = 3.293 + 1.307 u_p (km/s); for W, ρ_0 = 19.25 g/cm^3, U_s = 3.935 + 1.578 u_p (km/s).

Supplementary References

1. Jensen, B. J., Holtkamp, D. B., Rigg, P. A. & Dolan, D. H. Accuracy limits and window corrections for photon Doppler velocimetry. *J. Appl. Phys.* **101**, 013523 (2007).
2. Morard, G. *et al.* Melting of Fe–Ni–Si and Fe–Ni–S alloys at megabar pressures: implications for the core–mantle boundary temperature. *Phys. Chem. Miner.* **38**, 767-776 (2011).
3. Luo, S.-N. & Ahrens, T. J. Shock-induced superheating and melting curves of geophysically important minerals. *Phys. Earth Planet. Inter.* **143–144**, 369-386 (2004).
4. Zhang, Y. *et al.* Shock compression of Fe-Ni-Si system to 280 GPa: Implications for the composition of the Earth's outer core. *Geophys. Res. Lett.* **41**, 4554-4559 (2014).
5. Brown, J. M. & McQueen, R. G. Phase transitions, Grüneisen parameter, and elasticity for shocked iron between 77 GPa and 400 GPa. *J. Geophys. Res.* **91**, 7485-7494 (1986).
6. Brown, J. M., Fritz, J. N. & Hixson, R. S. Hugoniot data for iron. *J. Appl. Phys.* **88**, 5496-5498 (2000).
7. Duffy, T. S. & Ahrens, T. J. Sound velocities at high pressure and temperature and their geophysical implications. *J. Geophys. Res.* **97**, 4503-4520 (1992).
8. Hu, J. *et al.* Sound velocity measurements of tantalum under shock compression in the 10-110 GPa range. *J. Appl. Phys.* **111**, 033511 (2012).
9. Hixson, R. S. & Fritz, J. N. Shock compression of tungsten and molybdenum. *J. Appl. Phys.* **71**, 1721-1728 (1992).
